# Early Reflections on the Therapeutic Effects of Mindfulness-Based Therapies in Adults with Autism and Suggestions for Future Research

**DOI:** 10.20900/jpbs20210013

**Published:** 2021-07-29

**Authors:** Broc A. Pagni, B. Blair Braden

**Affiliations:** College of Health Solutions, Arizona State University, Tempe, AZ 85287, USA

**Keywords:** mindfulness, autism, depression, anxiety, mood, stress, rumination, clinical trial, intervention, meditation

## Abstract

Emerging research suggests mindfulness-based therapies positively impact adults with autism spectrum disorder (ASD). However, questions concerning intervention active ingredients, the breadth and duration of impact, and psychological and neural mechanisms of change remain. Here we discuss what is known about mindfulness-based therapies in adults with ASD and offer suggestions for future research.

## INTRODUCTION

Mindfulness-based therapies (MBTs) are a set of interventions that teach a variety of meditation techniques with an overarching theme of continuous redirection of attention toward present moment experience [1]. Core practices taught in MBTs involve directing attention toward the sensations of the body and the arising of thoughts and emotions with a nonjudgmental and nonreactive attitude. The efficacy of MBTs in reducing depression and anxiety has been demonstrated in clinical [2,3] and nonclinical populations [4]. Autism spectrum disorder (ASD) is a neurodevelopmental disorder characterized by two symptom domains: (1) repetitive and restricted behaviors and interests and (2) deficits in social communication and interaction [5]. Individuals with ASD have high rates of co-occurring symptoms of depression and anxiety for which MBTs may offer therapeutic improvement. Within the last decade, MBTs have been applied to ASD, showing initial signs of efficacy [3]. These early studies also highlight opportunities to improve scientific rigor and elucidate therapeutic mechanisms behind MBTs.

## CO-OCCURRING DEPRESSION AND ANXIETY IN AUTISM

According to metanalyses, 40% of young individuals with ASD have clinical anxiety [6] and adults are estimated to experience depression at quadruple the rate of the general population [7]. There is some indication prevalence may be higher in those with higher intelligence, fewer social impairments, and who are older [8,9]. Co-occurring depression and anxiety may compound preexisting challenges with employment, independent living, and long-term relationships [10,11]. Moreover, co-occurring depression and anxiety in ASD are associated with greater suicidality [12], social withdrawal, catatonia, brooding, and rumination [13,14]. Despite these discrepancies, adults with ASD are less likely to receive psychosocial therapy compared to non-ASD adults with depression and anxiety [15]. They are also more likely to be prescribed multiple medications even in the absence of ASD-specific FDA-approved pharmacotherapies and poor therapeutic responses to current available options [15,16].

## PSYCHOLOGICAL MEDIATORS OF CO-OCCURRING DEPRESSION/ANXIETY IN ASD

Attempts to elucidate psychological mediators of greater depression and anxiety risk in ASD are ongoing and critical to the development of evidence-based treatments. Early investigations suggest depression risk in ASD may be linked to heightened emotional brooding [14] and altered self-reflection [17]. In non-ASD depressed individuals, higher rates of private and public self-consciousness are associated with worse depression, however in adults with ASD higher private self-consciousness is associated with lower rates of depression [18]. Speculatively, higher private self-consciousness in ASD may reflect greater self-awareness which is markedly impaired in ASD. This shift could be a prerequisite for improved mental health [19]. Sex differences in ASD may also play a role in depression and anxiety symptomatology. For example, women with ASD show links between brooding and depression which explain only 20.7% of depression variance, whereas in men a combination of brooding, reflection, and self-consciousness explain ~50% of depression variance. This finding suggests unidentified psychological mediators may underlie depression in women and warrants future investigation into mediators of anxiety in ASD. Beyond sex- and ASD-specific nuances, common psychological substrates such as brooding and rumination may contribute to depression and anxiety symptomatology given consistent links have been demonstrated across diverse populations [20,21].

In adults with ASD, greater internal state awareness predicts fewer depressive symptoms, suggesting emotional awareness deficits in ASD may in part contribute to depression and anxiety susceptibility. Researchers have proposed “psychological therapies that target this type of ruminative self-focused attention and capitalize on the open curious quest for self-knowledge may therefore prove effective interventions to treat depression in adults with ASD” [14]. MBTs may be well suited since instructions involve acceptance of physical, mental, and emotional states and developing a curious attitude toward the quality of present moment experience. Additionally, MBTs may facilitate the transition from a narrative self-focus to an experiential self-focus [22]. Such a shift may undermine ruminative thinking, enhance emotional awareness, and direct attentional resources toward bottom-up sensory experience, thereby expanding the bandwidth of incoming information [23].

## MINDFULNESS-BASED THERAPIES FOR ADULTS WITH ASD

**Figure 1.**
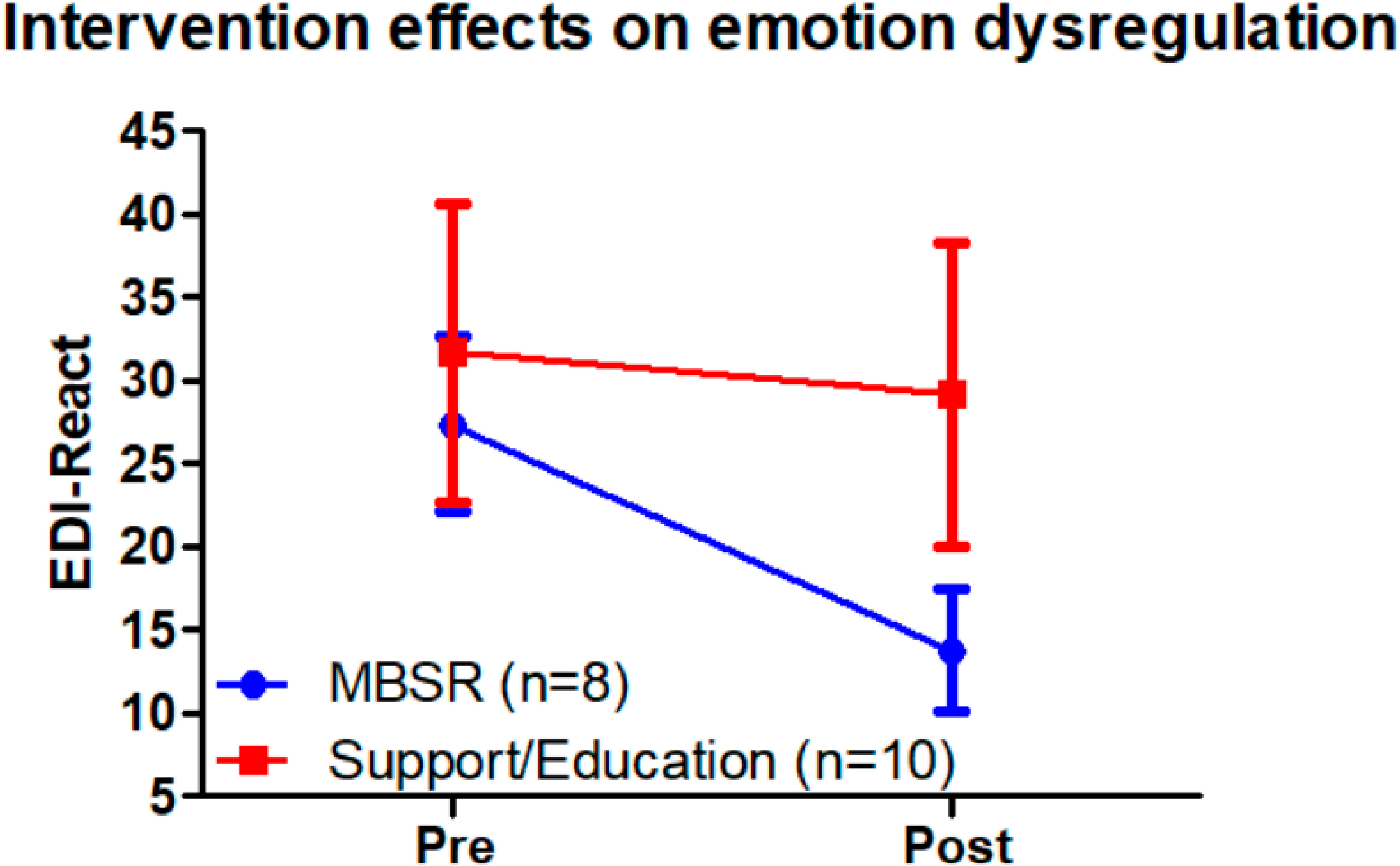
Group by time interaction demonstrating significant reduction in emotional reactivity in the MBSR, but not support/education, group.

Independent research groups have demonstrated MBTs, specifically Mindfulness-based Cognitive Therapy (MBCT) [3,24] and Mindfulness-based Stress Reduction (MBSR) [17,25], are efficacious for treating depression and anxiety in adults with ASD using various paradigms and comparison groups [26]. Intervention fidelity and feasibility standards have been confirmed to be high in adults with ASD, indicating no issues of concern for retention, material comprehension, participation, and intervention satisfaction [27]. MBTs may be beneficial across a range of psychological, emotional, and cognitive dimensions of wellbeing beyond depression and anxiety in adults with ASD. For example, MBSR has been shown to improve quality of life in adults with ASD by multiple groups [27], with our research showing improvements above and beyond an active control intervention [17]. This finding stands in contrast with an MBT metanalysis in other populations, showing that although MBTs have better mental health outcomes compared to other psychosocial interventions, improvements on quality of life, somatic, and social measures were equivalent. We have also found MBSR improves emotional regulation, compared to a social support/relaxation education (support/education) intervention, as evidenced by the subscale ‘Reactivity’ on the Emotional Dysregulation Inventory (Figure 1, EDI-react) [28]. However, these findings are preliminary and should be confirmed with larger samples. Other groups have found MBT-elicited improvements in agoraphobia, somatization, inadequacy in thinking, interpersonal distrust and sensitivity, and autism symptoms [3,25]. Together, these findings suggest MBT may have broad spectrum therapeutic effects that warrant further exploration.

MBT studies in adults with ASD have employed various comparison groups, intervention protocols/durations, outcome measures, and instructor-delivered procedures, making comparisons between studies difficult. For example, in a wait-list control study, MBCT was adapted for adults with ASD and produced reductions in depression, anxiety, and rumination and increases in positive affect in adults with ASD with sustained improvements 9 weeks post-intervention [24]. However, when compared to other active groups, such as social support [17] and cognitive behavioral therapy (CBT) [25], other researchers have observed similar improvements using 8-week and 13-week intervention periods, rendering it unclear if mindfulness training itself is the active ingredient of symptom improvement. For example, it is possible the interventions act upon domain-specific psychological and neural mechanisms and can be additive. Alternatively, therapeutic changes may be domain-general and arise from social support, intervention compliance and adherence, and/or stress education. Additionally, intervention duration or “dose” required for clinically significant improvements are unclear. Lastly, differences in instructor qualifications and delivery exist between studies. For example, MBSR was delivered in our studies by a certified MBSR instructor and an ASD clinician whereas others have used two psychologists with 8-month training in MBT-delivered interventions for ASD.

## SUGGESTIONS FOR FUTURE WORK ON MBTS IN ASD

For MBT research in adults with ASD, careful attention toward study designs is warranted to disentangle mindfulness-specific effects, identify psychological mediators of therapeutic gains, and compare findings across studies. Further, MBTs may have differential outcomes and act upon different mechanisms in adults with ASD compared to other clinical populations given atypicalities in self-referential [29], sensory [30], and emotional processing [31,32]. Future research would benefit from (1) rigorous active control groups, (2) assessing dose-response curves throughout the intervention period, (3) examining psychological and neural mediators of therapeutic improvement, and (4) extending follow-up periods. Ultimately, comparing MBTs to active control interventions with a focus on psychological and neural mediators will provide novel insights into elevated depression and anxiety rates in ASD and may inform psychologically- and biologically-based treatment strategies for a multitude of behavioral outcomes.
